# Linkage and association analysis of GAW15 simulated data: fine-mapping of chromosome 6 region

**DOI:** 10.1186/1753-6561-1-s1-s23

**Published:** 2007-12-18

**Authors:** Pimphen Charoen, Joanna M Biernacka, Heather J Cordell

**Affiliations:** 1 University of Cambridge, Diabetes and Inflammation Laboratory, Department of Medical Genetics, CIMR, Addenbrookes Hospital, Cambridge, CB2 2XY, UK; 2 Institute of Human Genetics, Newcastle University, International Centre for Life, Central Parkway, Newcastle upon Tyne, NE1 3BZ, UK

## Abstract

We performed linkage and family-based association analysis across chromosomes 1–22 in Replicates 1–5 of the Genetic Analysis Workshop 15 simulated data. Linkage analysis was performed using the Kong and Cox allele-sharing test as implemented in the program Merlin. Association analysis was performed using the transmission/disequilibrium test (TDT). A region on chromosome 6 was consistently highlighted as showing significant linkage to and association with the disease trait. We focused in on this region and performed fine-mapping using stepwise regression approaches using the case/control and family-based data. In this region, we also applied several new methods, implemented in the computer programs LAMP and Graphminer, respectively, that have recently been proposed for association analysis with family and/or case/control data. All methods confirmed the highly significant associations previously observed. Differentiating between potentially causal single nucleotide polymorphisms (SNPs) and other non-causal loci (associated with disease merely due to linkage disequilibrium) proved to be problematic. However, in most replicates we did identify two SNPs (either SNPs 3437 and 3439 from the dense SNP set, or SNPs 153 and 3437 from the combined non-dense/dense SNP set) that together explain most of the observed disease association in the DR/C locus region, and an additional SNP (3931 or 3933) that accounts for the association 5 cM away at locus D.

## Methods

We analyzed Replicates 1–5 of the Genetic Analysis Workshop 15 (GAW15) simulated data using both linkage and association methods. The analyses were performed without knowledge of the 'answers', however we subsequently obtained the 'answers' to inform our discussion. Using 1500 fully genotyped affected-sib-pair (ASP) families (parents and two children) in each replicate, we first tested for linkage across the genome using the Kong and Cox [1] exponential model allele-sharing test as implemented (in the form of a LOD score) in the program Merlin [2]. We then performed transmission/disequilibrium tests (TDT) [3] for association across the genome using the non-dense 9187 single-nucleotide polymorphism (SNP) set. Both affected sibs from a sibship were used, with non-independence between them accounted for by use of a robust 'information sandwich' variance estimator.

Given the highly significant results obtained on chromosome 6, we attempted to fine-map this region using first the original non-dense SNP set, then the dense chromosome 6 SNP set, and finally the combined non-dense/dense SNP set. We used the stepwise conditional logistic regression (case/pseudocontrol) approach for family data proposed by Cordell and Clayton [4]. We investigated whether any of the most significant loci could individually account for all of the association, and performed forward, backward, and forward then backward stepwise regression (using a *p*-value of 10^-3 ^to enter/exit the model) with the subset of SNPs showing a TDT χ^2 ^> 30. We repeated the regression analyses using logistic (rather than conditional logistic) regression in a case/control data set constructed by taking the first affected sib from each ASP together with the 2000 fully genotyped population controls provided.

In addition to fine-mapping, we also investigated the use of a new likelihood-based association analysis approach in this region [5] in which data from different family structures and unrelated controls are analyzed together. This approach is implemented in the program LAMP  and can be used to analyze either the full sample of ASPs together with the unrelated controls, the ASPs alone, a single affected sibling drawn from each ASP, or a single affected sibling together with the unrelated controls. Finally, we also used a recently proposed Bayesian approach for case/control association analysis [6] implemented in the program Graphminer. Although not specifically designed for fine-mapping, in simulation studies this approach has been shown to provide good localization of an underlying disease-causing variant, in comparison to more standard methods [6].

## Results

In each replicate, regardless of whether microsatellite or non-dense SNP markers were used, the most significant region of linkage was found on chromosome 6, centered around the 50 cM location where the 'true' DR and C loci reside. The Kong and Cox [1] LOD scores on chromosome 6 were consistently in range 70–90, whereas on other chromosomes, only weak evidence of linkage (LOD scores in the range 1–2, none of which was consistent between the five replicates) was found.

**Figure 1:**
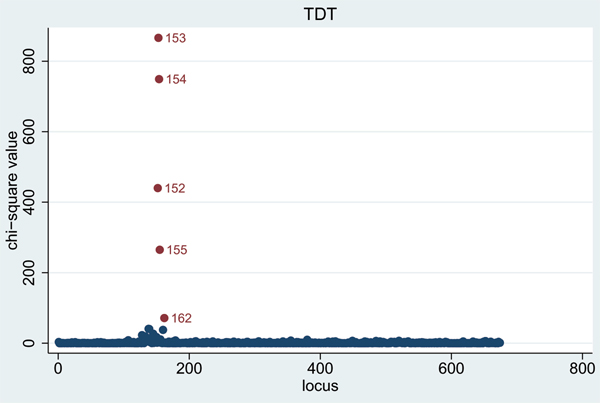
TDT results for non-dense SNPS on chromosome 6 (Replicate 1).

**Figure 2:**
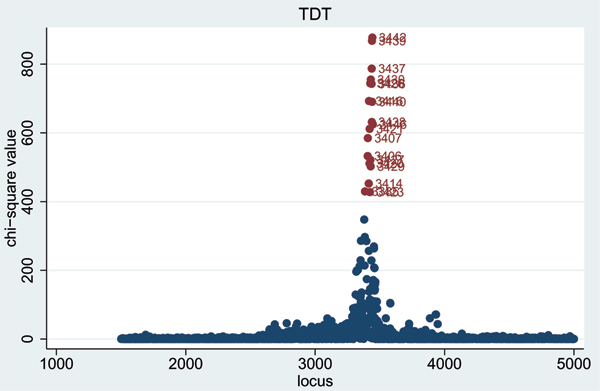
TDT results for dense SNPS on chromosome 6 (Replicate 1).

When using the non-dense SNPs, highly significant TDT [3] results were obtained in each replicate on chromosome 6 (see results for Replicate 1 in Figure 1), at SNPs 152–155 (all of which lie within 0.17 cM of 'true' loci DR and C) and at SNP 162 (which lies 0.05 cM from 'true' locus D). An even larger number of significant TDT results were found in this region when using the dense SNP set (Figure 2). The only other TDT results that were consistently significant (*p *< 10^-4^) across all five replicates were at SNP 389 on chromosome 11 (*p*-values 3 × 10^-23^, 1 × 10^-18^, 2 × 10^-15^, 1 × 10^-23^, 2 × 10^-21 ^in Replicates 1–5, respectively), and at SNP 269 on chromosome 18 (*p*-values 1 × 10^-7^, 5 × 10^-5^, 1 × 10^-7^, 1 × 10^-7^, 4 × 10^-9^, respectively). It is interesting to note from the 'answers' that the position of the chromosome 11 result corresponds to 'true' locus F which has an indirect effect on disease susceptibility through IgM, while the position of the chromosome 18 result corresponds to 'true' locus E.

**Figure 3:**
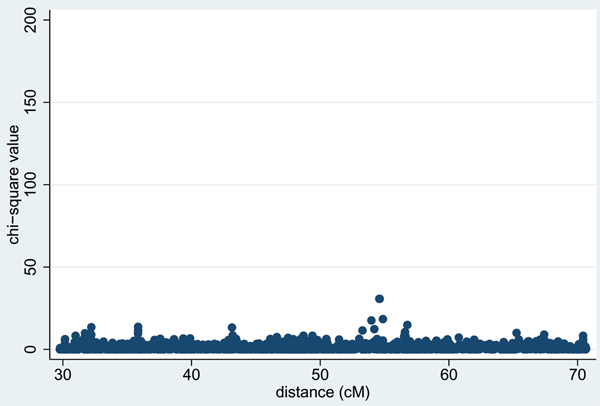
**Residual association after stepwise regression**. Results are shown after accounting for SNPs 3437 and 3439 (Replicate 1).

**Figure 4:**
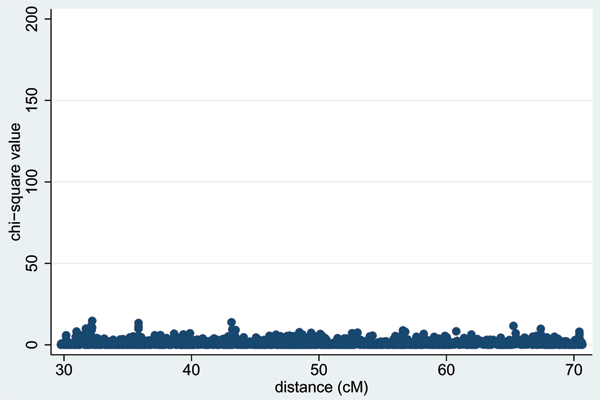
**Residual association after stepwise regression**. Results are shown after accounting for SNPS 3437, 3439 and 3931 (Replicate 1).

Table 1 shows the results from applying a stepwise regression procedure [4] for SNPs in the DR/C locus region in Replicates 1–5. With the non-dense SNPs, a number of significant loci remain in the final model, whereas with the dense SNP or combined non-dense/dense SNP set, only two or three SNPs (usually 3437 and 3439, or 3437 and 153) remain after forward then backward stepwise regression. These SNPs lie within 0.02 cM of the 'true' DR and C locus location (49.46 cM) and account for all the association at this location; however, once they were included in the model, some residual association (*p *= 4 × 10^-8^) was still seen at 'true' locus D (at 54.6 cM, Figure 3). Table 2 column 2 shows the SNPs around locus D that remained significant once SNPs in the DR/C locus region were included in the model. Applying a forward and backward stepwise regression procedure with these SNPs and those in the DR/C region generated a final model that generally included the two SNPs in the DR/C region and one (either 3931 or 3933) from the locus D region (Table 2, columns 3 and 4). In all except Replicate 2, no other SNPs across the chromosome showed significance once this combination of three SNPs was included in the model (Figure 4). Case/control logistic regression was found to give broadly similar results to the family-based regression analysis in terms of the magnitude and pattern of significance (data not shown).

**Table 1:** Forward and backward stepwise results for DR/C region^a^

Replicate	Non-dense SNPs		Dense SNPs			Non-dense + Dense SNPs combined		
			
	Forward	Backward	Forward	Backward	Forward then backward	Forward	Backward	Forward then backward
1	153	152	3442	3437	3437	3442	3437	3437
	154	153	3430	3439	3439	154	3439	3439
	162	154	3439			3439		
	155	155	3437			3437		
	152	162						
2	153	153	3442	3437	3437	3442	3437	3437
	154	154	3430	3439	3439	3437	3439	3439
	162	162	3437			3439		
	150		3439					
3	153	134	3442	3437	3437	3442	153	153
	154	153	3437	3439	3439	154	3437	3437
	162	154	3439			153	3439	
	134	162				3437		
	155							
4	153	138	3442	3437	3437	153	153	153
	154	139	3440	3439	3439	3437	3437	3437
	162	153	3439					
		154	3437					
		162						
5	153	153	3439	3437	3437	153	153	153
	154	154	3437	3439	3439	3437	3437	3437
	162	155						
	155	162						

^a^The SNPs listed are those that remain in the final model. SNPs in the 'Forward' columns are shown in the same order in which they are added to the model.

**Table 2:** Results from forwards and backwards stepwise procedure when adding SNPs in locus D region to SNPs in DR/C region

Replicate	SNPs included in model		SNPs remaining in model	
		
	DR/C region	D region	Forward	Backward
1	3437	162	3439	3437
	3439	3931	3437	3439
		3933	3931	3931
		3934		
2	3437	162	3439	3437
	3439	3931	3437	3439
		3933	3931	3931
		3934	3933	3933
			3934	3934
3	153	162	3439	153
	3437	3931	3437	3437
	3439	3933	3931	3931
		3934		
4	153	162	153	153
	3437	3931	3437	3437
		3933	3931	3933
		3934		
5	153	162	153	153
	3437	3931	3437	3437
		3933	3931	3933
		3934		

**Figure 5:**
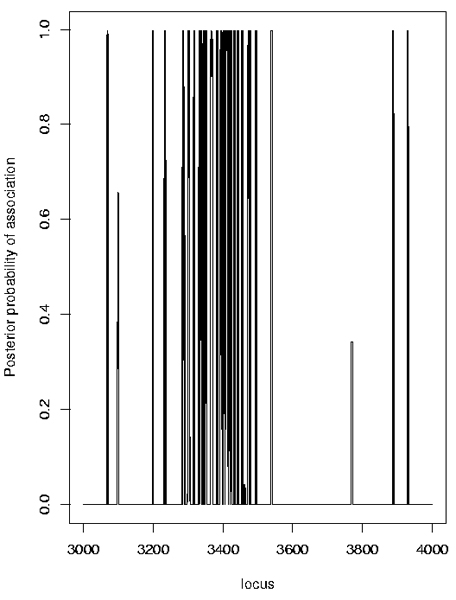
Graphminer results with dense SNPs (Replicate 1, λ = 10^-9^).

**Figure 6:**
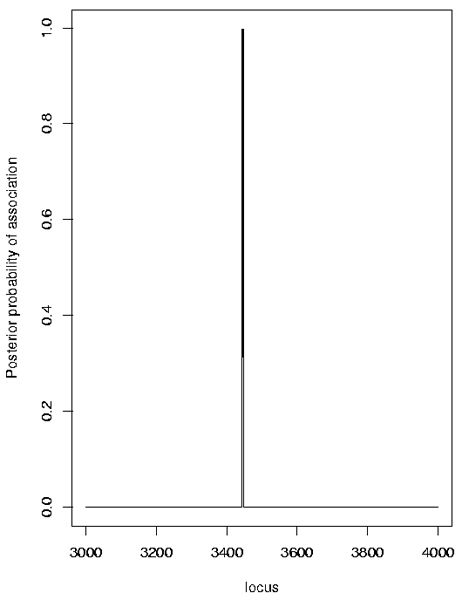
Graphminer results with dense SNPs (Replicate 1, λ = 10^-200^).

The results from the likelihood-based approach implemented in LAMP confirmed the association in this region. Not surprisingly, the highest significance with LAMP (a LOD score > 600) was found when using the maximum sample size (all ASPs together with all unrelated controls). Strong evidence for association was also found using the Bayesian approach implemented in Graphminer. Both the non-dense and the dense SNP sets (Figures 5, 6) provided strong evidence of association at one or more locations, but the results were quite sensitive to various Graphminer parameters, in particular the parameter λ, which represents the mean of the prior for the number of cliques (contiguous SNP sets) associated with disease. In the example distributed with the Graphminer program, λ = 10^-9 ^is suggested, but Verzilli et al. [6] used λ = 0.01. We investigated values of λ in the range 0.01–10^-300^. With larger λ values, localization of a single location was not achieved (Figure 5): the results mimic the single-locus results found with the TDT or logistic regression. With smaller λ values, a single location was achieved (Figure 6), but in some replicates this location did not always precisely correspond to the DR/C locus location (data not shown).

## Discussion

Not surprisingly, given the large sample size and strong simulated effects at the DR, C and D loci (mimicking known effects at HLA in diseases such as RA and type 1 diabetes), this chromosome 6 region was consistently significantly implicated in disease susceptibility via both linkage and association analysis. No other regions were consistently implicated by linkage analysis, but genome-wide association analysis detected SNPs associated with disease susceptibility at locations corresponding to 'true' loci E and F on chromosomes 18 and 11, in all five simulation replicates examined.

Fine mapping of the chromosome 6 region using Graphminer [6] did not always localize the true functional variants, and the choice of program parameters to use was not obvious. However, stepwise regression with the dense/combined SNP set was generally able to identify two SNPs (either 3437 and 3439 or 153 and 3437) that together account for all the association at the DR/C location, with residual evidence for association at locus D that can be accounted for through addition of a single SNP (either 3931 or 3933) in the locus D region. Using the genotype data provided in the 'answers', we determined that SNP 3437–3439 and 3437-153 haplotypes are in very high linkage disequilibrium (LD) with the causal DRB1-C haplotype, while SNPs 3931 and 3933 are in high LD with causal locus D. Therefore, it is not surprising that this combination of SNPs generally captured most of the observed association. Given the data available, statistically speaking, this is probably the limit of what can be achieved with regard to fine-mapping in this region. Investigation of other populations (with different LD patterns) might highlight the fact these identified SNPs merely tag the true causal effects. The next phase of a real study would most likely involve determination of all genetic variation in the region (e.g., through sequencing), followed by functional investigation of all potential causal variants identified.

## Competing interests

The author(s) declare that they have no competing interests.
